# Analysis of the predictive postoperative recurrence performance of three lymph node staging systems in patients with colon cancer

**DOI:** 10.3389/fonc.2025.1545082

**Published:** 2025-03-11

**Authors:** Ning Meng, Zhiqiang Wang, Yaqi Peng, Xiaoyan Wang, Wenju Yue, Le Wang, Wenqian Ma

**Affiliations:** ^1^ Department of General Surgery, Shijiazhuang People’s Hospital, Shijiazhuang, Hebei, China; ^2^ Basic College, Hebei Medical University, Shijiazhuang, Hebei, China; ^3^ Department of Endoscopy, The Fourth Hospital of Hebei Medical University, Shijiazhuang, China

**Keywords:** colon cancer, lymph node ratio (LNR), recurrence, nomogram, prognostic model

## Abstract

**Background:**

Colon cancer remains a major cause of cancer-related deaths worldwide, with recurrence post-surgery, posing a significant challenge. Accurate lymph node (LN) staging is critical for prognosis and treatment decisions, but traditional systems, such as the AJCC TNM, often fail to predict recurrence. This study compares the prognostic performance of three LN staging systems Lymph Node Ratio (LNR), Log Odds of Metastatic Lymph Nodes (LODDS), and pN in colon cancer.

**Methods:**

We retrospectively analyzed data from 812 colon cancer patients who underwent radical surgery at two tertiary hospitals (2010-2019). LNR, LODDS, and pN were calculated, and their ability to predict postoperative recurrence was assessed using C-index, AIC, BIC, and ROC curves. Machine learning models (LASSO, Random Forest, XGBoost) identified the most predictive staging system. A nomogram was developed integrating the best staging system with clinical factors to predict postoperative recurrence.

**Results:**

The study identified LNR as the most predictive staging system for colon cancer. The nomogram based on LNR, along with other variables such as T stage and tumor grade, demonstrated superior predictive performance compared to individual staging systems. In the training cohort, the nomogram achieved an AUC of 0.791 at 1 year, 0.815 at 3 years, and 0.789 at 5 years. The C-index for the nomogram was 0.788, higher than that of LNR (C-index = 0.694) and tumor stage (C-index = 0.665). The nomogram successfully stratified patients into high- and low-risk groups, with higher risk scores correlating with poorer survival outcomes. The validation cohort confirmed the robustness of the model, showing that patients with lower risk scores had better prognoses.

**Conclusions:**

LNR is an effective predictor of recurrence and prognosis in colon cancer. The nomogram developed from LNR and other clinical factors offers superior prognostication and can aid in personalized treatment strategies.

## Introduction

Colon cancer remains one of the leading causes of cancer-related morbidity and mortality worldwide (1–3). Despite advancements in surgical techniques and postoperative therapies, recurrence after surgery remains a significant challenge, especially for patients with postoperative recurrence or progression (4–6). Accurate staging of lymph nodes (LNs) after surgery is crucial for determining prognosis, guiding therapeutic decisions, and optimizing patient management (7–10). The staging of colon cancer is typically based on the extent of regional lymph node involvement, with several staging systems developed to assess the prognosis of patients post-surgery (11–13).

However, while these systems have provided valuable information, a number of critical issues remain unresolved (14, 15). First, there is a lack of consensus on the optimal staging system for predicting long-term outcomes in colon cancer patients, especially those with postoperative recurrence or atypical recurrence patterns (16). Traditional staging approaches, such as the American Joint Committee on Cancer (AJCC) TNM system, rely heavily on the number of positive LNs and their size (17). However, these parameters alone may not fully capture the complexity of metastasis in colon cancer (18). Recent research has suggested that certain lymph node characteristics, such as location, extranodal extension, and molecular features, may also play a crucial role in predicting the risk of recurrence (19, 20). Unfortunately, these factors are not consistently incorporated into existing staging systems.

A significant gap in previous research is the insufficient focus on lymph node staging systems in patients with recurrence after colon cancer surgery. Most studies have centered on early-stage or immediate post-surgery cases, overlooking the unique challenges posed by postoperative recurrence, which often presents with atypical features not captured by standard staging criteria (21, 22). Current systems AJCC, JSCCR, and NCCN were primarily designed for initial staging and may not effectively predict recurrence in patients with postoperative recurrence, as they often rely on parameters like lymph node size and number that may not reflect subtle changes during recurrence (18, 23, 24). Additionally, these systems vary slightly in criteria, leading to inconsistent prognostic outcomes. Few studies have comprehensively compared the predictive performance of these systems in recurrence cases, leaving a critical gap in understanding their clinical utility for this subset of patients (25). This study aims to address this by evaluating how well these staging systems predict prognosis in patients with colon cancer recurrence after surgery.

This study aims to analyze the postoperative predictive performance of three widely used lymph node staging systems—LNR (26, 27), LODDS (28, 29), and pN—in patients with colon cancer. By evaluating these systems’ ability to predict o postoperative recurrence in a large cohort, we seek to identify the most reliable method for stratifying risk in colon cancer patients and ultimately improve clinical decision-making. Additionally, we aim to develop a nomogram integrating the optimal lymph node staging system and other key clinical factors to provide a more personalized and accurate prognostic tool for patients, particularly those at high risk of recurrence.

## Materials and methods

### Patient selection

This study included patients who underwent radical colon cancer resection at Shijiazhuang People’s Hospital and the Fourth Hospital of Hebei Medical University between January 2010 and December 2019. The inclusion criteria were: 1) a pathologically confirmed diagnosis of colon adenocarcinoma; 2) age at diagnosis between 18 and 75 years. Patients were excluded based on the following criteria: 1) a history of other cancers or distant metastasis; 2) failure to undergo radical resection or having fewer than 12 lymph nodes harvested during surgery; 3) incomplete clinical or follow-up information (e.g., gender, age at diagnosis, tumor size, TNM stage, and CEA); 4) other histopathological types; 5) preoperative neoadjuvant chemotherapy or radiotherapy; 6) postoperative survival of less than 1 month; 7) tumors located at the rectosigmoid junction or rectum.

### Data collection

Clinical and pathological data were retrieved from the hospital’s medical database, including variables such as age, gender, marital status, carcinoembryonic antigen (CEA), TNM stage, T stage, N stage, tumor grade, tumor size, tumor location, number of harvested lymph nodes (ELN), number of metastatic lymph nodes (PLN), number of negative lymph nodes (NLN), number of tumor deposits (TD), follow-up duration, and follow-up status. Postoperative pathological results were independently reviewed by two experienced pathologists. In cases of diagnostic discrepancies, a third senior pathologist examined the samples. A final diagnosis was determined when at least two pathologists reached a consensus.

The lymph node ratio (LNR) was calculated as PLN/ELN (30), and the log odds of metastatic lymph nodes (LODDS) was calculated using the formula (31): log10((PLN+0.05)/(NLN+0.05)). The optimal cutoff values for continuous LNR and LODDS were determined using X-Tile software. LNR was categorized as <0.050, 0.051-0.300, and >0.301, while LODDS was categorized as <-2.45, -2.45 to -0.37, and >-0.37. Tumors located in the cecum, ascending colon, hepatic flexure, and transverse colon were classified as right-sided colon cancer, while tumors in the splenic flexure, descending colon, and sigmoid colon were classified as left-sided colon cancer (32, 33). TNM staging was determined according to the AJCC 8th edition. Our study’s primary endpoint was postoperative recurrence, defined as the time from tumor resection to postoperative recurrence.

### Treatment

All CC patients underwent radical resection. Chemotherapy with CAPOX or FOLFOX was introduced between 3 and 8 weeks after resection. According to the NCCN, all CC patients with stage III received eight cycles of chemotherapy.

### Follow-up

Patients were monitored every 3–6 months via outpatient visits and phone calls. The follow-up period began at diagnosis and continued until either the patient’s death or January 31, 2023. The follow-up information include the time of recurrence, the sites of recurrence and survival outcomes. It is recommended to perform enhanced CT scans of the chest, abdomen, and pelvis every six months during the first two years, followed by annual scans for the next three years. Additionally, annually endoscopy should be conducted after five years post-surgery to rule out local recurrence.

### Screening of prognostic factors

In this study, univariate Cox regression analysis was performed using the “survival” package in R software to examine the relationship between clinical-pathological variables and patient survival in the training set. The clinical-pathological variables included age, gender, marital status, T stage, N stage, tumor stage, lymph node ratio (LNR), log odds of metastatic lymph nodes (LODDS), primary tumor site, tumor size, histological grade, number of harvested lymph nodes (ELN), number of metastatic lymph nodes (PLN), number of negative lymph nodes (NLN), tumor deposits (TD), and carcinoembryonic antigen (CEA). Variables that were statistically significant in the univariate analysis were then included in a multivariate Cox regression analysis to further identify prognostic factors.

### Selection of the optimal LN staging system

To determine the optimal LN staging system, we first compared the prognostic abilities of three LN staging systems using the concordance index (C-index), Akaike Information Criterion (AIC), and Bayesian Information Criterion (BIC). Additionally, receiver operating characteristic (ROC) curves and the area under the curve (AUC) were generated to evaluate the predictive value of each system.

Finally, three machine learning algorithms: Least Absolute Shrinkage and Selection Operator (LASSO) regression (34), random forest (35), and extreme gradient boosting (XGBoost) (36), were applied to analyze each dimension. These methods directly select raw features without any linear combinations or transformations, and the selected features remain consistent with the original data. They provide feature importance scores, allowing for the assessment of each feature’s contribution to model prediction. This insight is valuable for feature selection and model interpretation. LASSO regression was performed using the “glmnet” R package’s LASSOCV module, XGBoost analysis was conducted using the “XGBoost” R package to extract feature importance, and random forest classification was trained on the training set generated by the “randomForestSRC” R package (37–39). Feature importance was extracted using the “feature importance” function.

### Construction of the nomogram

A nomogram is a graphical representation of a mathematical relationship, commonly used to estimate the results of a formula visually. In this study, a nomogram for predicting postoperative recurrence was constructed to estimate the prognosis of CC patients based on the optimal LN staging system. Survival curves, calibration plots, C-index, and ROC curves were plotted to assess the accuracy of the prognostic nomogram in both the training and validation cohorts.

### Statistical analysis

All analyses were performed using R software (version 4.4.1) and IBM SPSS (version 26). A P-value of <0.05 was considered statistically significant. The chi-square (χ²) test and t-test were used to examine relationships between categorical variables, while means and medians were calculated to summarize descriptive variables. Dummy variables were created for categorical data using one-hot encoding. Ordinal and interval variables were converted into numeric variables for analysis.

## Results

### Patient demographics and clinical characteristics

This study included a total of 812 colon cancer patients, comprising 549 patients from Hebei Medical University Fourth Hospital and 263 patients from Shijiazhuang People’s Hospital. Of the 549 patients from Hebei Medical University Fourth Hospital, the data were randomly split into a training set (n = 329) and an internal validation set (n = 220) using a 6:4 ratio, via the “caret” package in R software. The 263 patients from Shijiazhuang People’s Hospital were assigned to the external validation set. The follow-up duration for all patients ranged from 1 to 96 months, with a median follow-up time of 42 months. The recurrence time ranged from 1 to 90 months, with a median recurrence time of the training set, validation set, and external validation set were 19.4, 19.9 and 18.7 months respectively.

In the training set, postoperative recurrence occurred in 147 cases (44.6%), including 12 cases of local recurrence (8.2%), 17 cases of multiple organ metastases (11.6%), and 118 cases of single organ metastasis (80.2%). In the internal validation set, postoperative recurrence were observed in 101 cases (45.9%), with 7 cases of local recurrence (6.9%), 15 cases of multiple organmetastases (14.9%), and 79 cases of single organ metastasis (78.2%). In the external validation set, postoperative recurrence occurred in 114 cases (43.3%), including 13 cases of local recurrence (11.4%), 10 cases of multiple organ metastases (8.8%), and 91 cases of single organ metastasis (79.8%) (**Table 1**).

**Table 1:** Baseline demographic and clinicopathological features of the patients.

Variable	Training set1	Internal validation	External validation	P
	(N=329)	(N=220)	(N=263)	
Age (Years), Mean	63.4 (6.76)	63.4 (6.81)	62.9 (6.69)	0.998
Gender				
Female	144 (43.8%)	88 (40.0%)	121 (46.0%)	0.431
Male	185 (56.2%)	132 (60.0%)	142 (54.0%)	
Maritalstatus				
Married	192 (58.4%)	134 (60.9%)	160 (60.8%)	0.612
Unmarried	137 (41.6%)	86 (39.1%)	103 (39.2%)	
CEA				
negative/normal	196 (59.6%)	135 (61.4%)	169 (64.3%)	0.741
positive/elevated	133 (40.4%)	85 (38.6%)	94 (35.7%)	
Primary tumor site				
Left	131 (39.8%)	82 (37.3%)	111 (42.2%)	0.610
Right	198 (60.2%)	138 (62.7%)	152 (57.8%)	
Tumor size(mm), Mean	46.9 (24.7)	44.3 (23.9)	48.4 (22.0)	0.217
<5cm	201 (61.1%)	140 (63.6%)	142 (54.0%)	0.609
≥5cm	128 (38.9%)	80 (36.4%)	121 (46.0%)	
LNR, Mean (SD)	0.085(0.155)	0.088 (0.156)	0.0751 (0.138)	0.661
<0.050	207 (62.9%)	133 (60.5%)	168 (63.9%)	0.810
0.051-0.300	89 (27.1%)	65 (29.5%)	74 (28.1%)	
>0.301	33 (10.0%)	22 (10.0%)	21 (8.0%)	
LODDS, Mean (SD)	-1.79 (0.996)	-1.76 (1.02)	-1.84 (0.948)	0.757
<-2.450	163 (49.5%)	106 (48.2%)	130 (49.4%)	0.945
-2.45 to -0.37	133 (40.4%)	92 (41.8%)	111 (42.2%)	
>-0.37	33 (10.1%)	22 (10.0%)	22 (8.4%)	
TNM Stage				
I	71 (21.6%)	54 (24.5%)	44 (16.7%)	0.485
II	106 (32.2%)	61 (27.8%)	108 (41.1%)	
III	152 (46.2%)	105 (47.7%)	111 (42.2%)	
Grade				
I	55 (16.7%)	35 (15.9%)	40 (15.2%)	0.641
II	171 (52.0%)	114 (51.8%)	157 (59.7%)	
III	63 (19.1%)	50 (22.7%)	48 (18.3%)	
IV	40 (12.2%)	21 (9.6%)	18 (6.8%)	
pT Stage				
T1/2	90 (27.4%)	69 (31.4%)	76 (28.9%)	0.358
T3/4	239 (72.6%)	151 (68.6%)	187 (71.1%)	
pN Stage				
N0	177 (53.8%)	115 (52.3%)	152 (57.8%)	0.843
N1	100 (30.4%)	72 (32.7%)	68 (25.9%)	
N2	52 (15.8%)	33 (15.0%)	43 (16.3%)	
ELN, Mean (SD)	21.7 (9.85)	22.1 (10.3)	20.1 (8.16)	0.643
NLN, Mean (SD)	20.0 (9.99)	20.3 (10.5)	21.7 (8.15)	0.685
PLN, Mean (SD)	1.73 (3.34)	1.77 (3.39)	1.64 (2.97)	0.703
TD, Mean (SD)	0.252 (0.823)	0.191 (0.689)	0.240 (1.17)	0.345
Postoperative recurrence				
Negative	182(55.4%)	119(54.1%)	149(56.7%)	0.852
Positive	147(44.6%)	101(45.9%)	114(43.3%)	

**Figure 1:**
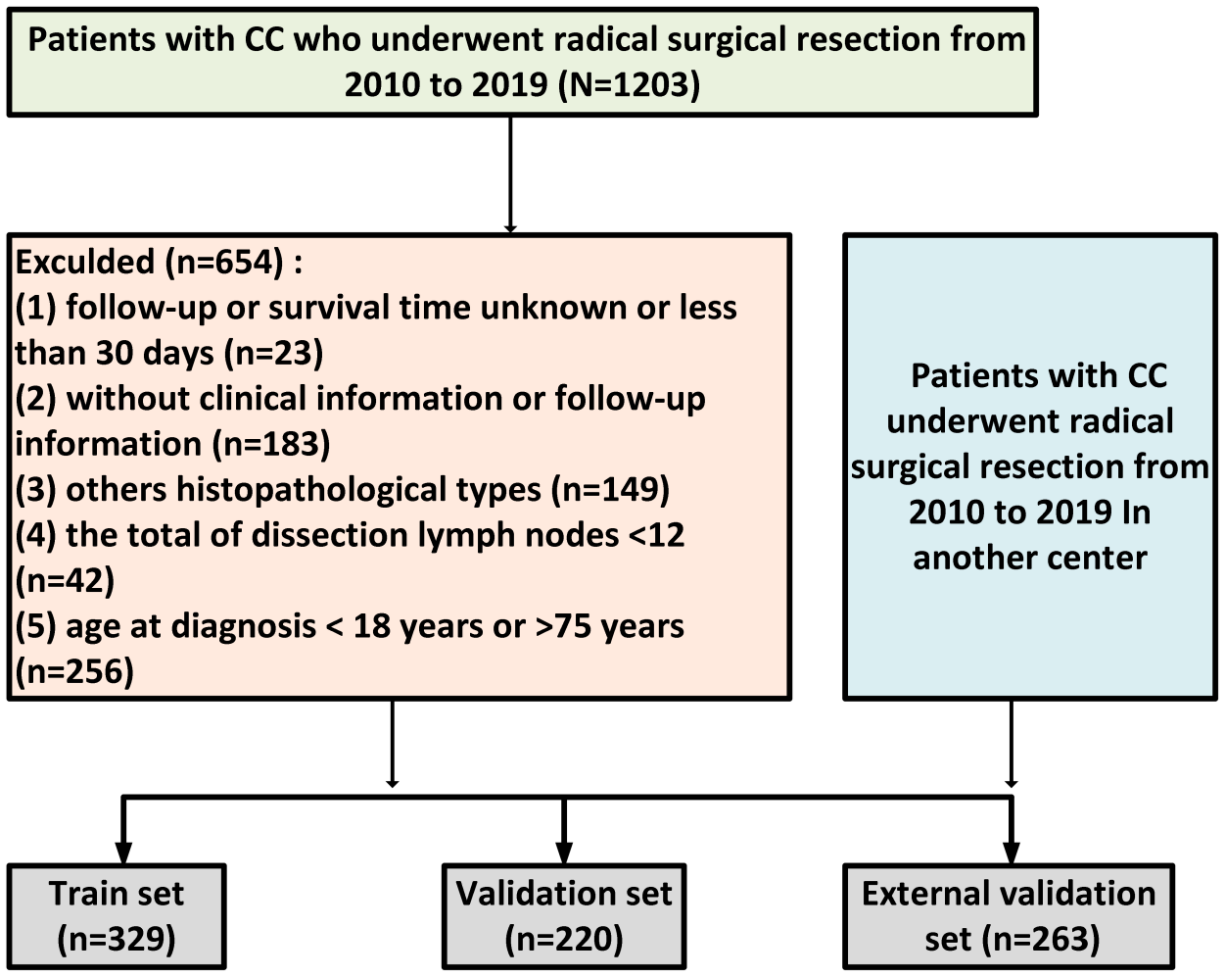
the flowchart of patients cohort selection.

A detailed flowchart is shown in **Figure 1**. All participants were diagnosed between the ages of 18 and 75, with a median age of 63.4 (SD 6.77) years. The median ages of the training set, validation set, and external validation set were 63.4 (SD 6.76), 63.4 (SD 6.81), and 62.9 (SD 6.69) years, respectively. Interestingly, most patients were diagnosed between the ages of 60 and 69. Additionally, each patient had a minimum of 12 lymph nodes dissected, with median values of 21.7 (SD 9.85), 22.1 (SD 10.3), and 20.1 (SD 8.16) for the training set, internal validation set, and external validation set, respectively. No significant differences were observed between the two validation sets and the training set in terms of clinical factors, as detailed in **Table 1**.

### Identification of prognostic clinical factors for postoperative recurrence

Univariate Cox regression analysis revealed significant associations between survival time and several clinical variables, including T stage, N stage, tumor stage, histological grade, LNR, LODDS, CEA, and tumor deposits (TD). The estimated regression coefficients and hazard ratios (HR) for each variable are detailed in **Table 2**. Notably, both continuous LNR and LODDS demonstrated statistically significant hazard ratios of 23.312 (95% CI: 10.201–53.275, p < 0.001) and 1.965 (95% CI: 1.631–2.367, p < 0.001), respectively, indicating their strong prognostic value. A multivariate Cox regression analysis was performed to further assess the associations between pN stage, LODDS, LNR, and disease free survival (c) in CC patients. The results indicated that LODDS, LNR, and pN status significantly influenced the postoperative recurrence of CC patients (**Table 3**).

**Table 2:** Univariate analysis for postoperative recurrence in the training set.

Variable	Univariate	
	HR (95% CI)	*P*
Age(years)		
≤50	Reference	
>50	0.725(0.299-1.758)	0.477
Gender		
Female	Reference	
Male	0.794(0.648-1.466)	0.901
Tumor size		
<5cm	Reference	
≥5cm	1.271(0.841-1.921)	0.255
pT Stage		
T1/2	Reference	
T3/4	4.184(2.198-7.965)	<0.001
TNM Stage		
I	Reference	
II	2.208(1.004-4.853)	0.048
III	5.211(2.559-10.610)	<0.001
Grade		
I	Reference	
II	2.968(1.402-6.283)	0.004
III	4.363(1.932-9.856)	<0.001
IV	3.892(1.705-8.887)	0.001
Maritalstatus		
Married	Reference	
Unmarried	1.266(0.816-1.842)	0.327
Primary tumor site		
Left	Reference	
Right	2.087(1.317-2.308)	0.002
CEA		
negative/normal	Reference	
positive/elevated	2.437(1.608-3.692)	<0.001
LNR		
<0.050	Reference	
0.051 to 0.300	2.673(1.659-4.308)	<0.001
>0.301	8.180(4.812-13.908)	<0.001
LODDS		
<-2.450	Reference	
-2.45 to -0.37	2.632(1.616-4.288)	<0.001
>-0.37	9.425(5.329-16.670)	<0.001
pN Stage		
N0	Reference	
N1	2.406(1.480-3.913)	<0.001
N2	5.273(3.138-8.859)	<0.001
TD	1.353(1.130-1.619)	<0.001
ELN	0.983(0.959-1.006)	0.147
PLN	1.115(1.076-1.156)	<0.001
NLN	0.952(0.924-0.980)	<0.001

**Table 3:** Association of pN stage, LNR, and LODDS with postoperative recurrence in the training cohort.

Variable	Multivariate analysis					
	HR (95% CI)	*P*	HR (95% CI)	*P*	HR (95% CI)	*P*
pT Stage						
T1/2	Reference		Reference		Reference	
T3/4	2.809 (1.426-5.531)	0.002	2.614 (1.320-5.177)	0.006	2.662 (1.347-5.263)	0.004
Grade						
I	Reference		Reference		Reference	
II	2.344 (1.089-5.047)	0.029	2.366 (1.096-5.106)	0.028	2.180 (1.017-4.669)	0.045
III	3.301 (1.426-7.643)	0.005	2.752 (1.176-6.439)	0.019	2.578 (1.109-5.966)	0.028
IV	4.809 (2.057-11.240)	<0.001	4.809 (2.057-11.240)	<0.001	4.321 (1.856-10.063)	<0.001
Primary tumor site						
Left	Reference		Reference		Reference	
Right	2.405 (1.508-3.835)	<0.001	2.364 (1.477-3.783)	<0.001	2.360 (1.475-3.777)	<0.001
CEA						
negative/normal	Reference		Reference		Reference	
positive/elevated	1.756 (1.135-2.717)	0.011	1.735 (1.117-2.695)	0.014	1.738 (1.738-2.697)	0.013
**TD**	1.070 (0.875-1.307)	0.511	1.078 (0.886-1.311)	0.451	1.058 (0.870-1.286)	0.576
pN Stage						
N0	Reference					
N1	2.096 (1.279-3.436)	0.003				
N2	4.220 (2.397-7.429)	<0.001				
LODDS						
<-2.450			Reference			
-2.45 to -0.37			2.358 (1.435-3.874)	<0.001		
>-0.37			6.034 (3.246-11.217)	<0.001		
LNR						
<0.050					Reference	
0.051 to 0.300					2.349 (1.451-3.804)	<0.001
>0.301					5.339 (2.989-9.537)	<0.001

### Selection of the optimal LN staging system

**Figure 2:**
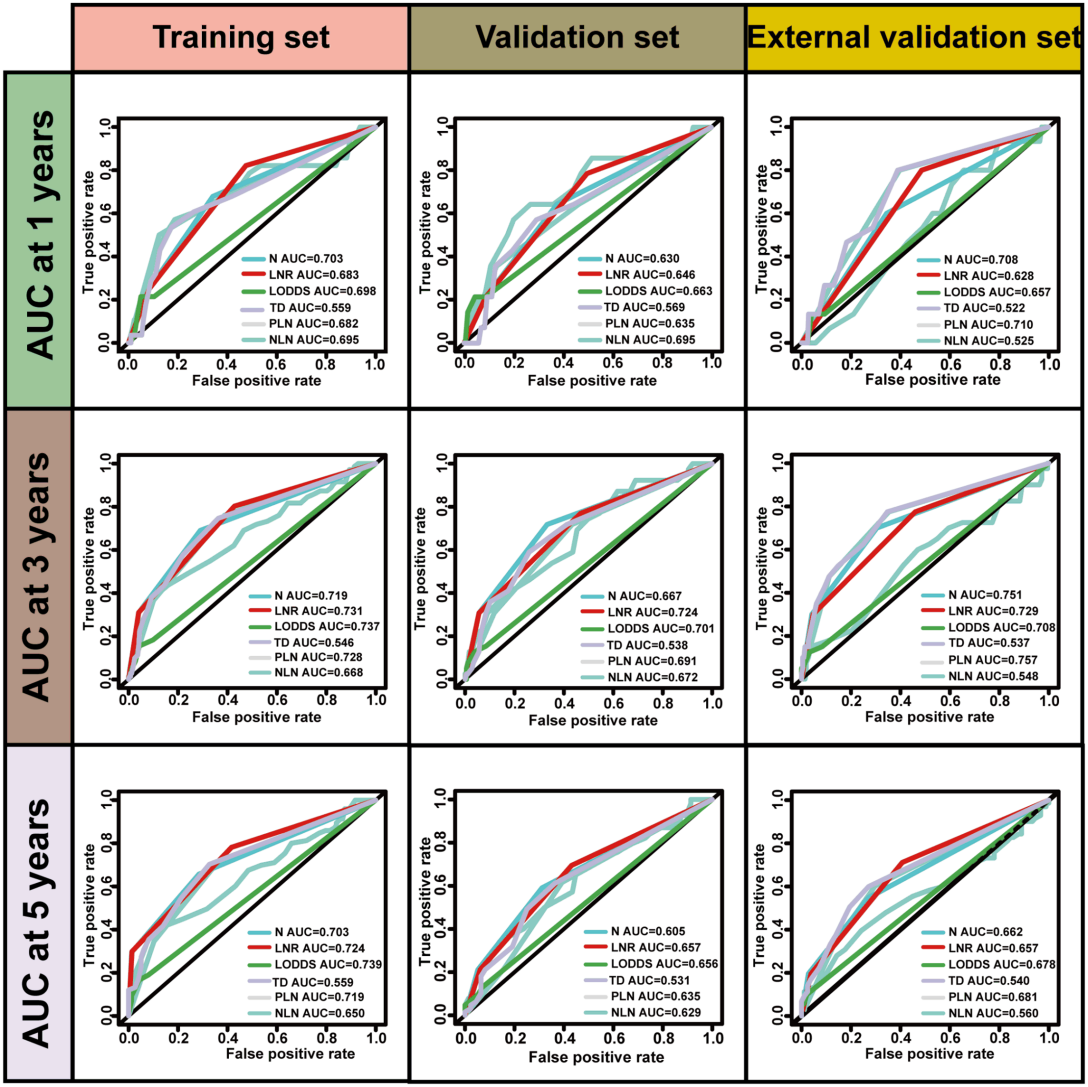
ROC curves for predicting postoperative 1-, 3-, and 5-year recurrence in three sets.

No significant differences were observed in the prognostic predictive abilities of the three lymph node (LN) staging methods across the training, validation, and external validation cohorts (**Table 4**). In the training cohort, the C indices for LNR, LODDS, and pN were 0.694, 0.701, and 0.685, respectively, while in the validation cohort, the C indices were 0.665, 0.653, and 0.623. In the external validation cohort, the C indices were 0.658, 0.655, and 0.676, respectively. Additionally, the AIC values for each system in the training cohort were 961.664, 961.418, and 976.831, while in the validation cohort, they were 621.956, 626.118, and 635.452; in the external validation cohort, the AIC values were 835.556, 837.433, and 839.737. To further explore the prognostic prediction ability of the nomogram, AUC values were plotted, as shown in **Figure 2**. In all three cohorts, no significant differences were observed in the time-dependent 1-year, 3-year, and 5-year AUCs for LNR, LODDS, and pN (P > 0.05). These findings suggest that the discriminative quality of the three systems is similar, which aligns with the previously mentioned results. Therefore, machine learning methods, including LASSO, XGBoost, and RF, were employed to further identify the optimal LN staging system in terms of predictive ability. The analysis included the following variables: LNR, LODDS, pN, tumor grade, CEA, TD, PLN, ELN, NLN, primary tumor site, and tumor size.

**Table 4:** Prediction performance of the three lymph nodal staging systems for postoperative recurrence.

Variable	C-index (95% CI)	AIC	BIC
Training set			
LNR	0.694(0.667-0.720)	961.664	966.751
LODDS	0.701(0.676-0.726)	961.418	966.505
pN Stage	0.685(0.657-0.712)	976.831	981.917
Internal Validation			
LNR	0.665(0.632-0.699)	621.956	626.335
LODDS	0.653(0.620-0.687)	626.118	630.497
pN Stage	0.623(0.588-0.658)	635.452	639.831
External validaton			
LNR	0.658(0.629-0.687)	835.556	840.488
LODDS	0.655(0.626-0.684)	837.433	842.365
pN Stage	0.676(0.647-0.705)	839.737	844.669

**Figure 3:**
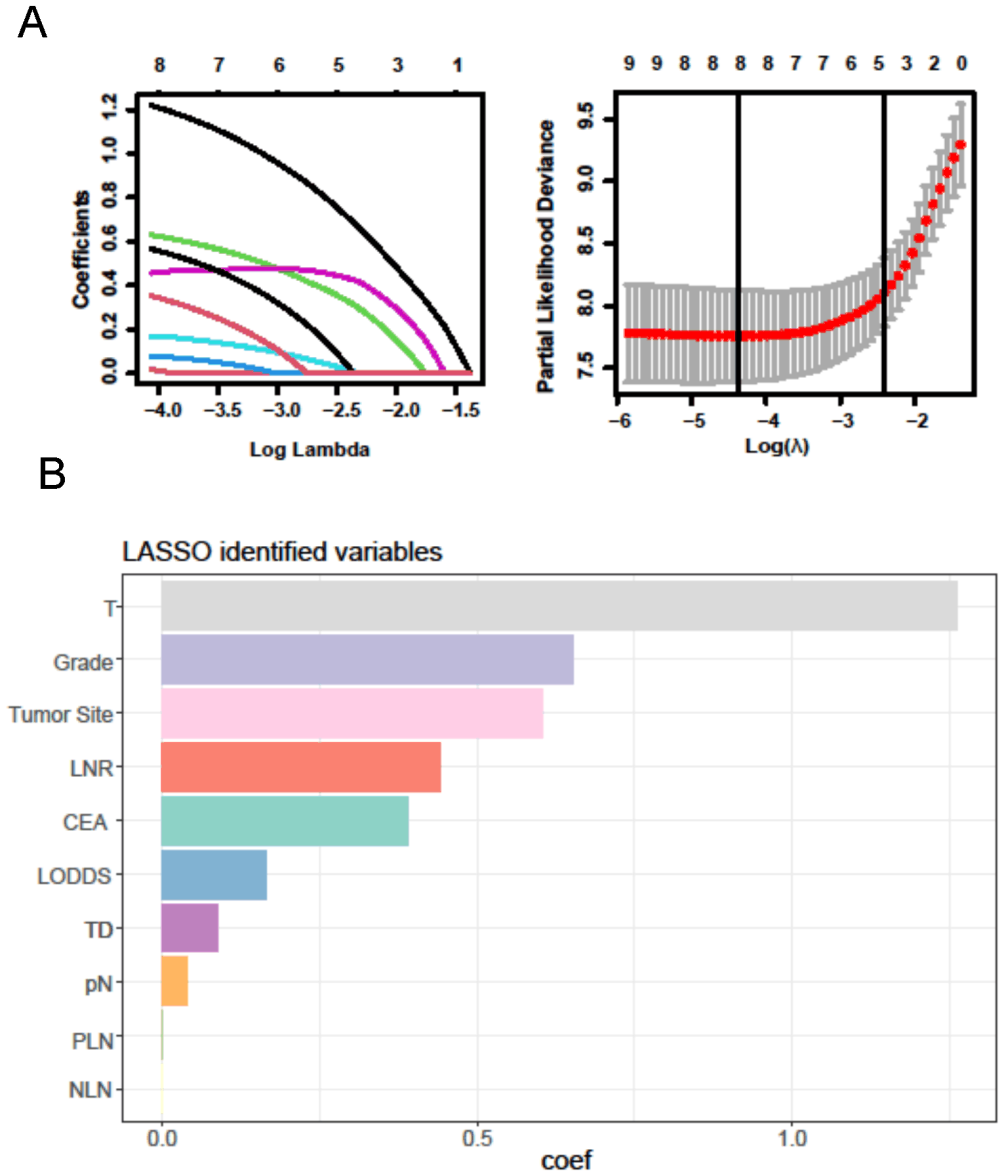
LASSO regression analysis. **(A)** LASSO regression to identify the optimal variable. **(B)** The coefficients of each variable in LASSO analysis.

**Figure 4:**
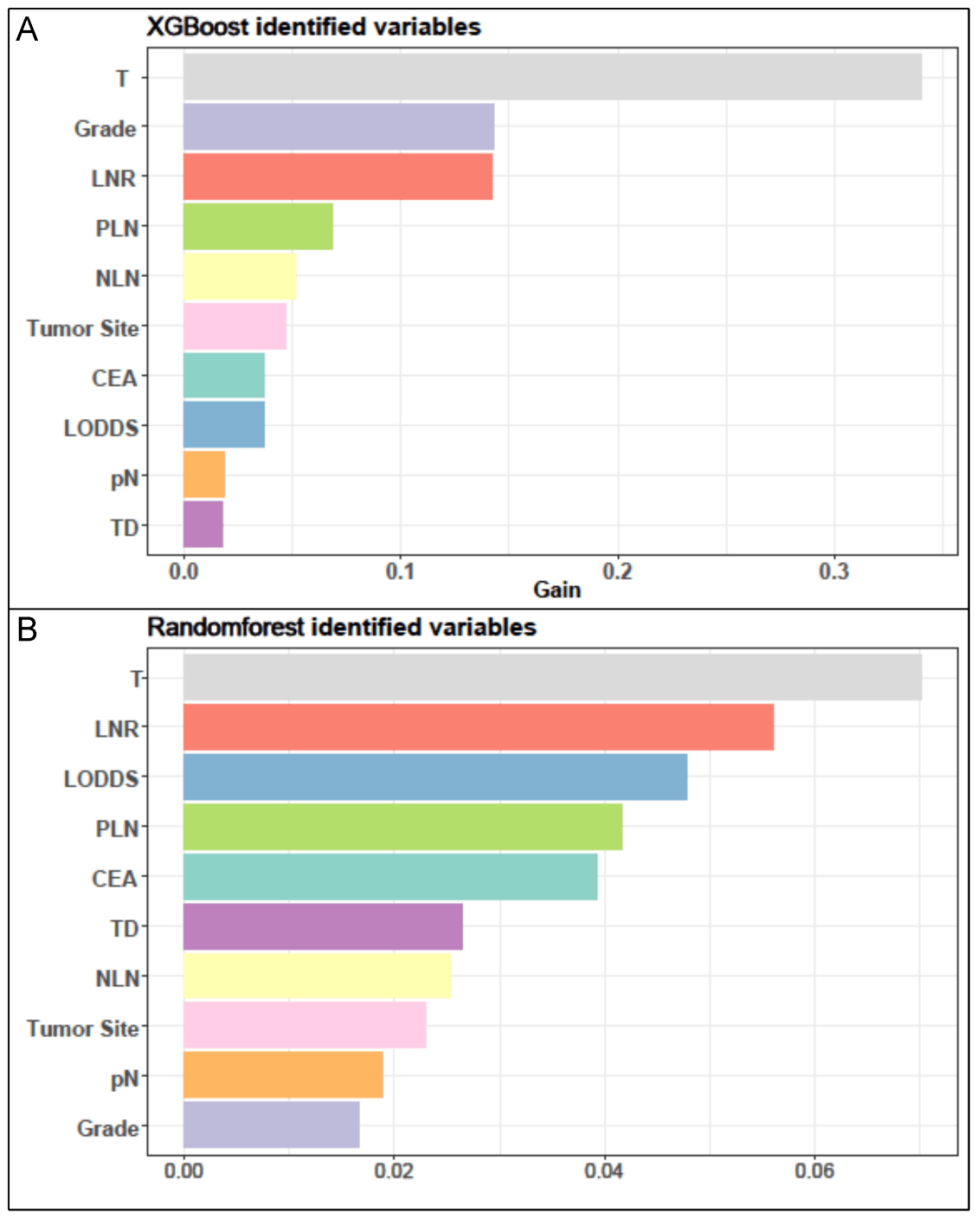
The results of XGBoost and RF analyses. **(A)** The feature importance in XGBoost analysis. **(B)** the importance score of features in RF analysis.

For the LASSO regression analysis, 10-fold cross-validation was performed, and the optimal α parameter (α = 0.001) was adjusted to control the strength of regularization. Features with a coefficient value of 0 were excluded (**Figure 3A**). The importance of the features was determined by the absolute values of the coefficients obtained from the final LASSO model fitted to the training cohort (**Figure 3B**). Feature importance in the LASSO model was inferred from the magnitude of the coefficients. The T stage had the largest coefficient, followed by tumor grade. Among the three lymph node staging systems, the LNR coefficient was the largest, suggesting that LNR is one of the most important features. Subsequently, XGBoost was performed on the training dataset. The importance values for each variable are shown in **Figure 4A**. Similar to the LASSO results, the T stage and tumor grade were identified as the most important features, with LNR also showing high importance. Feature importance from the Random Forest analysis is shown in **Figure 4B**. Based on the results from all three machine learning methods, we conclude that LNR is the most predictive and influential feature among the three lymph node staging systems.

### Development and validation of the LNR-based nomogram

**Figure 5:**
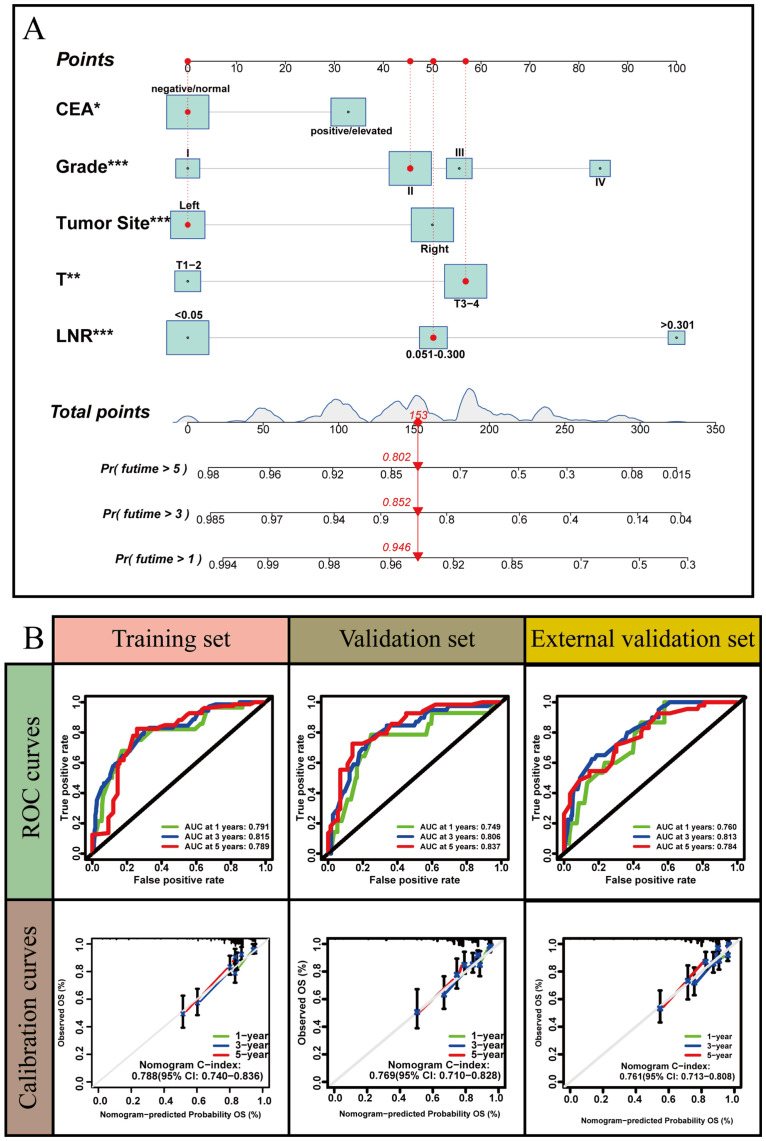
A nomogram based on LNR staging. **(A)** The nomogram was built based on five clinical variables in the training set. **(B)** The ROC curves, calibration curves and C-index values for predicting postoperative 1-, 3-, and 5-year recurrence. *: P<0.05, **: P<0.01, *** :P<0.001.

**Figure 6:**
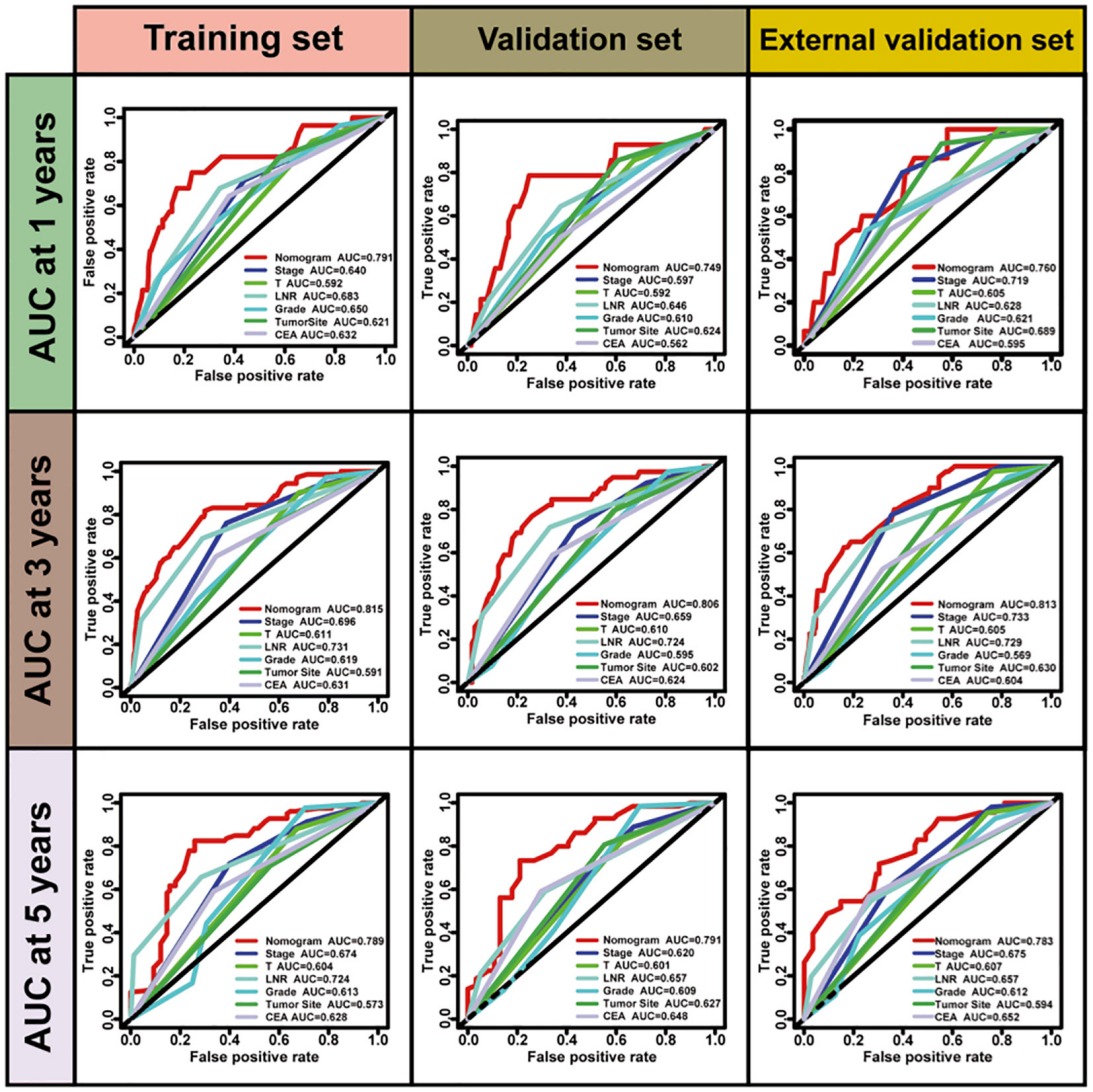
The ROC curves of nomogram for predicting postoperative recurrence compared with other clinical variables.

**Figure 7:**
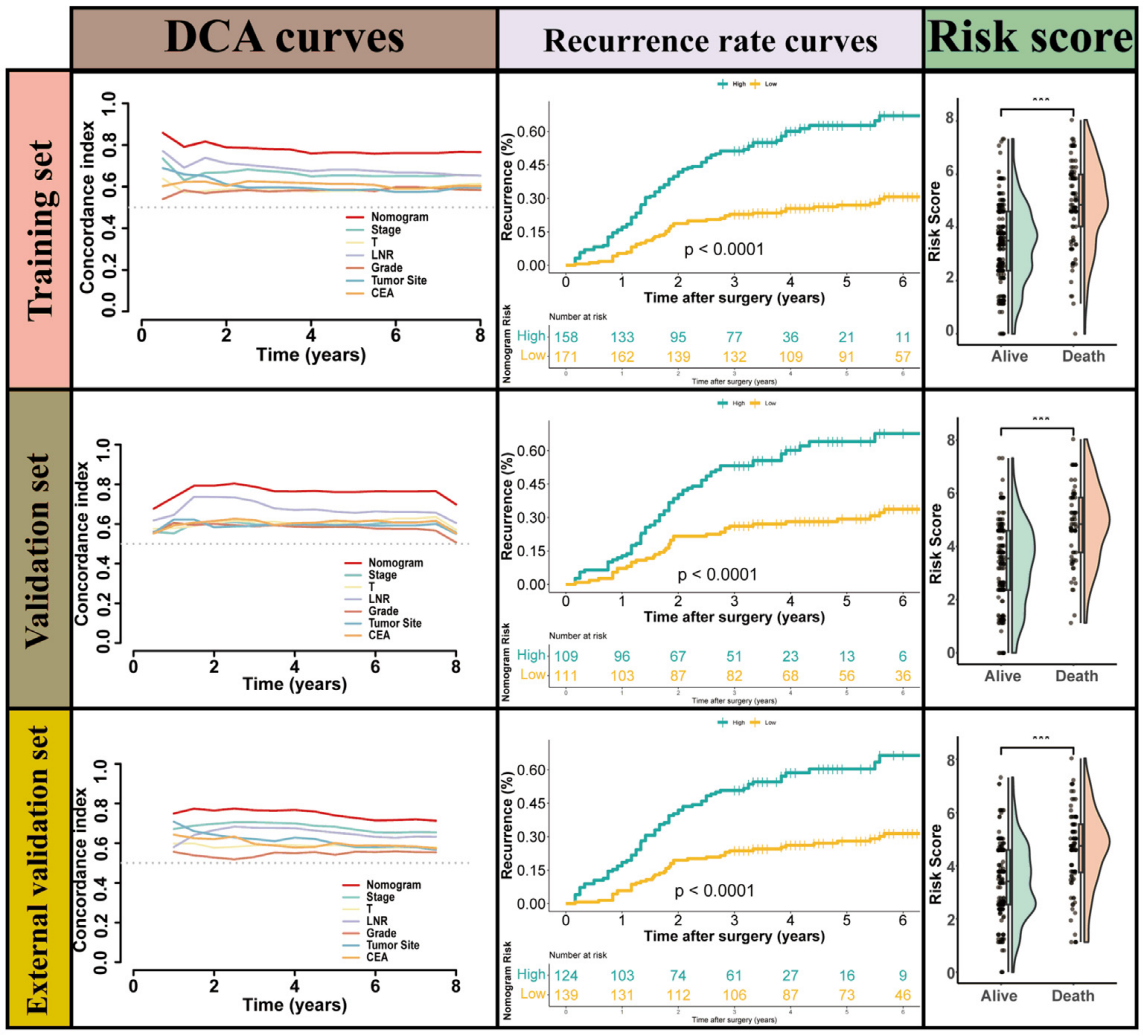
the performance of Nomogram for predicting postoperative recurrence and the cumulative recurrence rate stratified by the nomogram s scores using a Mantel–Haenszel’s Hazard Ratio (MHR) test.

LNR was selected as the optimal LN staging system to develop a new nomogram for estimating the postoperative recurrence of CC patients (**Figure 5A**). Other prognostic variables, including T stage, tumor grade, tumor location, and CEA, were also incorporated. In the nomogram, each variable is represented by a vertical scale, indicating its value range. By aligning the values of these variables and observing the intersection points on the nomogram, we can estimate the 1-year, 3-year, and 5-year postoperative recurrence probabilities. Calibration curves, ROC curves, and time-dependent C-index curves were then plotted to assess the predictive performance of the nomogram (**Figures 5**–**7**), demonstrating its good applicability and accuracy. In the training cohort, the nomogram’s AUC was superior to that of other variables, with AUCs of 0.791 at 1 year, 0.815 at 3 years, and 0.789 at 5 years (**Figure 6**). The nomogram’s C-index was 0.788, higher than that of LNR (C-index = 0.694) and tumor stage (C-index = 0.665) (**Figure 7**). Additionally, based on the median risk scores calculated from the nomogram, patients in the training cohort were classified into high-risk and low-risk groups. Patients with higher nomogram risk scores had higher postoperative recurrence rate than those with lower scores (**Figure 7**). The nomogram risk scores for patients with different recurrence statuses are shown in **Figure 7**, indicating that higher risk scores correlate with increased recurrence rate in CC patients. Furthermore, the same risk scores derived from the nomogram formula were applied to the validation cohort, where patients with lower risk scores had better prognoses than those with higher scores (**Figure 7**). These results suggest that the nomogram can accurately and conveniently predict the postoperative recurrence of CC patients.

## Discussion

This study aimed to assess the postoperative predictive performance of three commonly used lymph node staging systems LNR, LODDS, and pN in patients with colon cancer who experienced recurrence. Our results demonstrated that while there were no significant differences in the predictive abilities of the three systems across training, validation, and external validation cohorts, machine learning techniques identified the LNR as the most predictive and influential feature. Furthermore, the development of a nomogram based on LNR, along with other key clinical factors such as T stage, tumor grade, tumor location, and CEA, proved to be a robust tool for estimating the prognosis of patients with postoperative recurrence. The nomogram outperformed the individual staging systems in terms of C-index and AUC, suggesting that incorporating multiple prognostic factors into a comprehensive tool can improve survival prediction accuracy. Additionally, our findings indicate that higher nomogram risk scores correlated with poorer survival outcomes, suggesting the utility of this nomogram for patient stratification in clinical practice.

Several studies have explored the role of lymph node staging in colon cancer prognosis, but few have focused on recurrence (40, 41). Traditional staging systems like the AJCC TNM system have been widely utilized in colon cancer prognostication. However, these systems primarily rely on the number and size of metastatic lymph nodes, which may not adequately capture the complexities of postoperative recurrence (42). Some research have highlighted the limitations of TNM staging in predicting recurrence, especially in patients with subtle or atypical metastasis patterns (43, 44). They emphasized the importance of including additional factors, such as extranodal extension and molecular characteristics, to enhance the predictive power of staging systems (30). Similarly, our study found that LNR, which accounts for the ratio of metastatic to total lymph nodes, provided better prognostic discrimination, particularly in cases of recurrence. This is consistent with findings by Pei et al. who reported that LNR had superior predictive value compared to the AJCC TNM system in colon cancer patients with lymph node involvement (45). In our study, the incorporation of LNR into a nomogram led to more accurate survival predictions, further supporting its clinical relevance.

Further comparison with studies that utilized the LODDS system also demonstrates the promise of this variable in colon cancer prognosis. The LODDS, which adjusts for both the number of positive and negative lymph nodes, has shown improved prognostic value over traditional systems. Previous studies have consistently found that LODDS provided more refined risk stratification than simple lymph node counts (46, 47). Our findings align with these reports, as LODDS was identified as a significant prognostic factor in our univariate and multivariate Cox regression analyses. However, when compared to LNR, LODDS did not demonstrate superior predictive capability in our study. This suggests that while LODDS is a valuable parameter, LNR may still provide a more straightforward and clinically applicable measure of lymph node involvement in patients with recurrence.

Despite its strengths, this study has several limitations that warrant discussion. First, the retrospective nature of the study introduces potential biases, such as incomplete follow-up data and unmeasured confounding factors. Although we adjusted for various clinical and pathological variables, other unknown factors could have influenced our results. Additionally, the study was conducted at two tertiary hospitals, which may limit the generalizability of our findings to broader patient populations. Further multi-center, prospective studies with larger sample sizes are needed to validate the predictive performance of the LNR-based nomogram in diverse clinical settings. Another limitation is the lack of molecular data, which could have provided additional insights into the mechanisms behind recurrence and further refined the prognostic model. Future studies should aim to integrate genomic and molecular profiling with traditional staging systems to develop more comprehensive tools for patient stratification. Moreover, while our analysis demonstrated the superiority of the LNR-based nomogram over individual staging systems, the utility of this tool in guiding treatment decisions and improving patient outcomes remains to be fully evaluated. Large-scale clinical trials assessing the impact of nomogram-guided treatment strategies on patient survival would provide valuable evidence for its clinical implementation.

In conclusion, this study highlights the utility of the lymph node ratio (LNR) in predicting the prognosis of colon cancer patients with recurrence after surgery. By incorporating LNR into a nomogram alongside other clinical factors, we developed a robust tool for patient stratification, which outperforms individual staging systems in terms of accuracy and predictive ability. The findings support the importance of refined lymph node staging systems, particularly in the context of recurrence, and emphasize the need for more personalized treatment approaches. Although this nomogram shows promise, further validation in larger and more diverse cohorts, along with the integration of molecular and genomic data, is required to optimize its clinical utility. Ultimately, this research paves the way for more effective prognostication and treatment strategies, enhancing the management of colon cancer patients at high risk of recurrence.

## Data Availability

The raw data supporting the conclusions of this article will be made available by the authors, without undue reservation.
